# Systemic therapy with pemigatinib and sintilimab followed by resection for recurrent FGFR-2-positive intrahepatic cholangiocarcinoma: a case report

**DOI:** 10.3389/fonc.2025.1527372

**Published:** 2025-04-04

**Authors:** Yuchen Yang, Jingfeng Li, Di Ma, Fengjie Hao, Weixia Li, Jing Xie, Lihan Qian, Junqing Wang, Yongjun Chen

**Affiliations:** ^1^ Departement of General Surgery, Ruijin Hospital Shanghai Jiaotong University School of Medicine, Shanghai, China; ^2^ Department of Radiology, Ruijin Hospital, Shanghai Jiao Tong University School of Medicine, Shanghai, China; ^3^ Department of Pathology, Ruijin Hospital, Shanghai Jiaotong University School of Medicine, Shanghai, China

**Keywords:** intrahepatic cholangiocarcinoma, FGFR-2, systemic therapy, locally advanced recurrence, case report

## Abstract

**Background:**

Fibroblast growth factor receptor-2 (FGFR-2) mutations are frequently observed in intrahepatic cholangiocarcinoma (ICC). While FGFR2-targeted therapies are primarily studied in advanced ICC, this report presents a rare case of locally recurrent ICC treated with systemic therapy, leading to significant tumor regression and successful R0 resection.

**Case presentation:**

A 51-year-old female underwent right posterior hepatectomy and cholecystectomy in 2018 for ICC. In August 2022, postoperative MRI revealed tumor recurrence near the hepatic vein, accompanied by intrahepatic bile duct dilation and a tumor thrombus. Given the tumor’s proximity to critical structures and confirmed FGFR-2 fusion, systemic therapy with pemigatinib and sintilimab was initiated. After four cycles, the tumor showed partial remission, with a reduction in the bile duct tumor thrombus. In May 2023, the patient underwent successful right hemi-hepatectomy. Postoperatively, she continued combination therapy without recurrence or metastasis for 19 months.

**Conclusion:**

This case highlights the efficacy of pemigatinib-based systemic therapy in achieving tumor regression and enabling curative resection in locally recurrent FGFR-2-positive ICC. The successful outcome underscores the potential of targeted therapies in managing recurrent ICC, warranting further investigation.

## Introduction

Intrahepatic cholangiocarcinoma (ICC) is a highly aggressive epithelial tumor characterized by rapid progression and a generally poor prognosis (1, 2). ICC recurs frequently post-resection, with surgery as the main cure. For patients with recurrent tumors diagnosed in a timely manner and with adequate residual liver volume, surgery is still recommended (3). Nevertheless, it remains uncertain whether preoperative systemic therapy, primarily medication-based, can improve long-term survival in recurrent ICC, as no definitive research conclusions exist. FGFR-2 inhibitors like pemigatinib are emerging for advanced cases, but their use in recurrent ICC before surgery is rare (4).Thus, neoadjuvant therapy for cholangiocarcinoma has not yet been fully established.

Currently, systemic treatment regimens for ICC are largely based on research findings in advanced ICC. The main systemic treatments include chemotherapy, immunotherapy, and targeted therapy (5). Over the past decade, the standard first-line treatment for advanced ICC has been a combination of gemcitabine and cisplatin, though this approach offers limited efficacy and is associated with severe adverse events (6). Recent breakthroughs in targeted and immunotherapy research have provided new treatment strategies for ICC (7). Moreover, specific molecular alterations, such as fibroblast growth factor receptor-2 (FGFR-2) fusions and isocitrate dehydrogenase 1 (IDH1) mutations, have led to the development of targeted therapies as second-line treatments in some clinical studies (8).

In this study, we present a rare case of recurrent ICC with a bile duct tumor thrombus. To our knowledge, instances of recurrent ICC treated with systemic therapy followed by curative resection are infrequently documented. In this case, the patient underwent a comprehensive treatment strategy involving pemigatinib and sintilimab as systemic therapy, followed by surgical resection. Postoperatively, the patient continued on pemigatinib and sintilimab, and after 19 months of follow-up, there has been no evidence of tumor recurrence.

## Case presentation

This patient is a 51-year-old female who presented with right upper quadrant pain after a large meal. No other significant clinical symptoms were reported. The patient has an old surgical scar on the upper abdomen, and there are no other notable physical findings. Her medical history includes an abdominal surgery five years ago at an external medical facility, during which a right hepatic tumor resection and cholecystectomy were performed. Pathological analysis at that time confirmed ICC, with the largest tumor measuring 4 cm and no evidence of lymph node involvement or distant metastasis. According to the American Joint Committee on Cancer (AJCC) staging system, the tumor was classified as pT2N0M0, corresponding to Stage II. Immunohistochemical findings were as follows: Alpha-fetoprotein (AFP) (-), Glypican 3 (-), Hepar-1 (focal +), Arginase-1 (-), Cytokeratin 19 (CK19) (+), Carbohydrate antigen 19-9 (CA19-9) (+), CDX-2 (-), Villin (+), Cytokeratin 7 (CK) (+), Cytokeratin 20 (CK20) (-). The genetic testing of polymerase chain reaction (PCR) indicates that the tumor is microsatellite stable (MSS).

During the initial diagnosis in our hospital, the laboratory analysis revealed elevated levels of protein induced by vitamin K absence or antagonist (PIVKA). Liver magnetic resonance imaging (MRI) identified an irregular signal lesion in the right posterior hepatic lobe, measuring approximately 27 x 19 mm. The lesion was located near the middle hepatic vein, raising concerns of tumor recurrence at the previous surgical site. Additionally, intraductal carcinoma emboli were observed in the local bile duct. A PET-CT scan from the local hospital confirmed the absence of distant metastasis.

After multidisciplinary discussions involving the radiology and hepatobiliary surgery departments at our hospital, the patient was diagnosed as local recurrent ICC. It was determined that there were no contraindications for surgery, making resection feasible. However, given the patient’s recurrent ICC and the lack of regular adjuvant therapy following the initial surgery due to the corona virus disease 2019, there is a substantial risk of postoperative recurrence. Furthermore, the tumor’s proximity to the middle hepatic vein and the presence of an intraductal tumor thrombus, along with its close association with the common bile duct, complicate the surgical approach. Preserving the middle hepatic vein may be difficult during surgery, necessitating an extended right hemi-hepatectomy, which increases the risk of postoperative hepatic failure.

Considering these factors, we decided to apply systemic therapy as the primary treatment option. On January 13, 2023, the patient underwent a CT-guided percutaneous liver biopsy, followed by genetic testing. Histopathological analysis confirmed the recurrence of ICC, and the next generation sequencing revealed a fusion mutation involving the FGFR2 gene. Pemigatinib was then applied as the target therapy with sintilimab as the immunotherapy. Consequently, the patient was initiated on systemic therapy, which included the administration of sintilimab (200 mg) on Day 1 and oral pemigatinib (13.5 mg) daily for 14 days, followed by a 7-day rest period. Laboratory parameters following each treatment cycle are provided in **Supplementary Table 1**.

**Figure 1:**
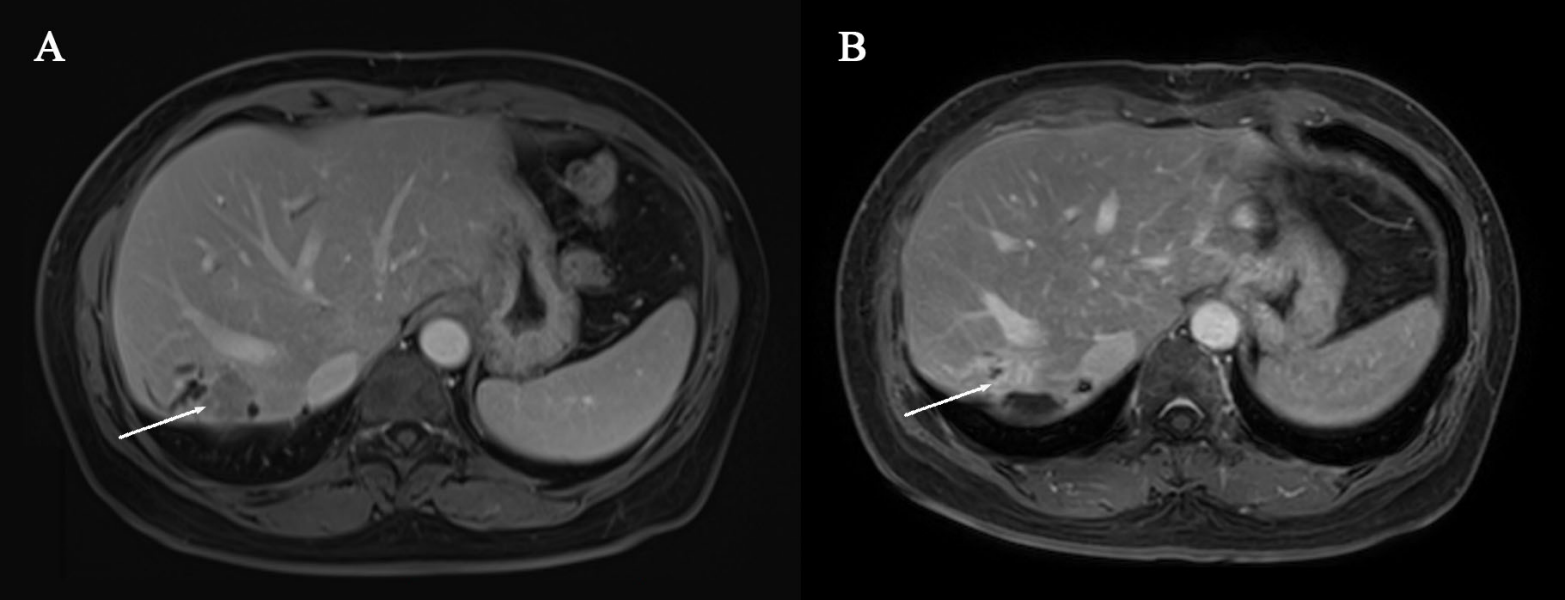
**(A)** Pre-systemic therapy enhanced-contrast MRI image during venous phase **(B)** Post-systemic therapy enhanced-contrast MRI image during venous phase. Arrow indicates tumor in **(A, B)**.

**Figure 2:**
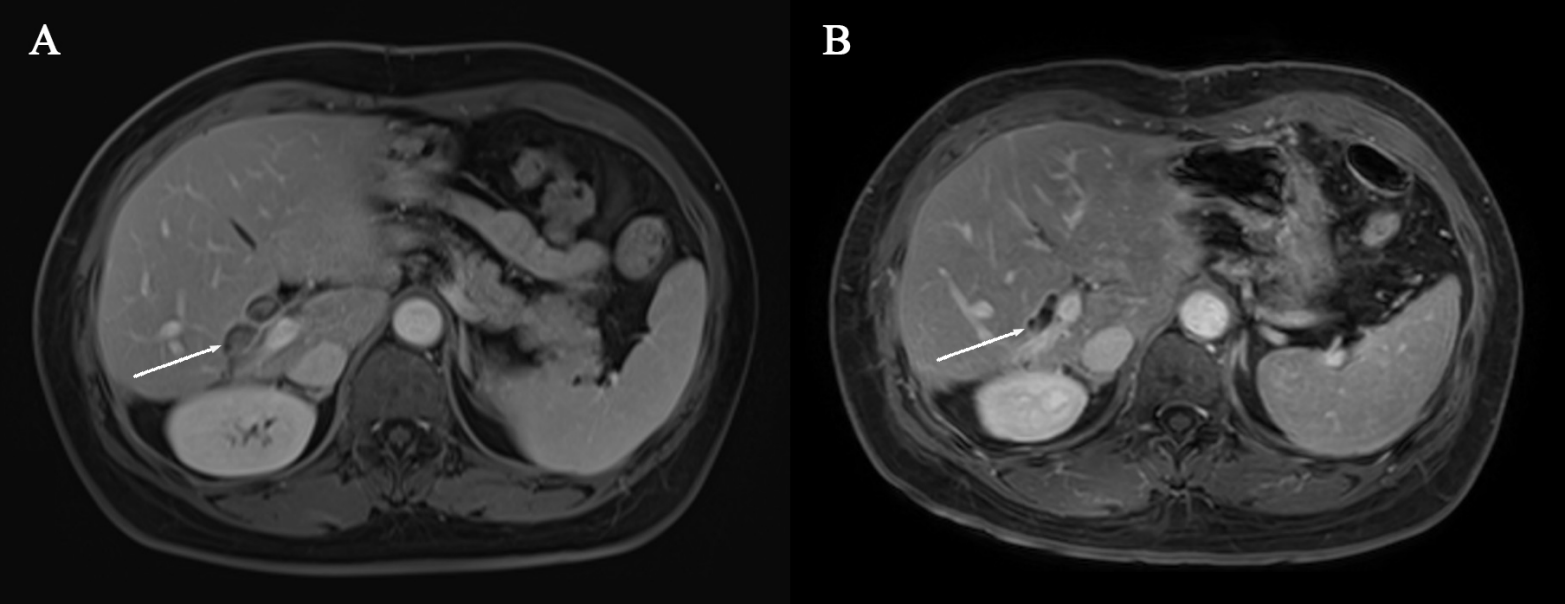
**(A)** Pre-systemic therapy enhanced-contrast MRI image during portal phase **(B)** Post-systemic therapy enhanced-contrast MRI image during portal phase. Arrow indicates biliary duct carcinoma thrombosis in **(A, B)**.

During the course of treatment, the patient reported an improvement in pain compared to pre-treatment levels. Only mild adverse events (grade 1–2) were observed, including hair loss and dry mouth, with no other treatment-related adverse events noted which is consistent with the results of previous studies (9). MRI images before and after systemic therapy are displayed in **Figures 1**, **2**.

After four treatment cycles, a reassessment of the tumor was conducted. MRI revealed a reduction in tumor size by over 30% compared to the initial evaluation, indicating partial remission (**Figure 1**). Additionally, there was a reduction in the intraductal carcinoma emboli within the bile duct (**Figure 2**). Following another multidisciplinary discussion, a residual right hemi-hepatectomy was performed on May 8, 2023.

**Figure 3:**
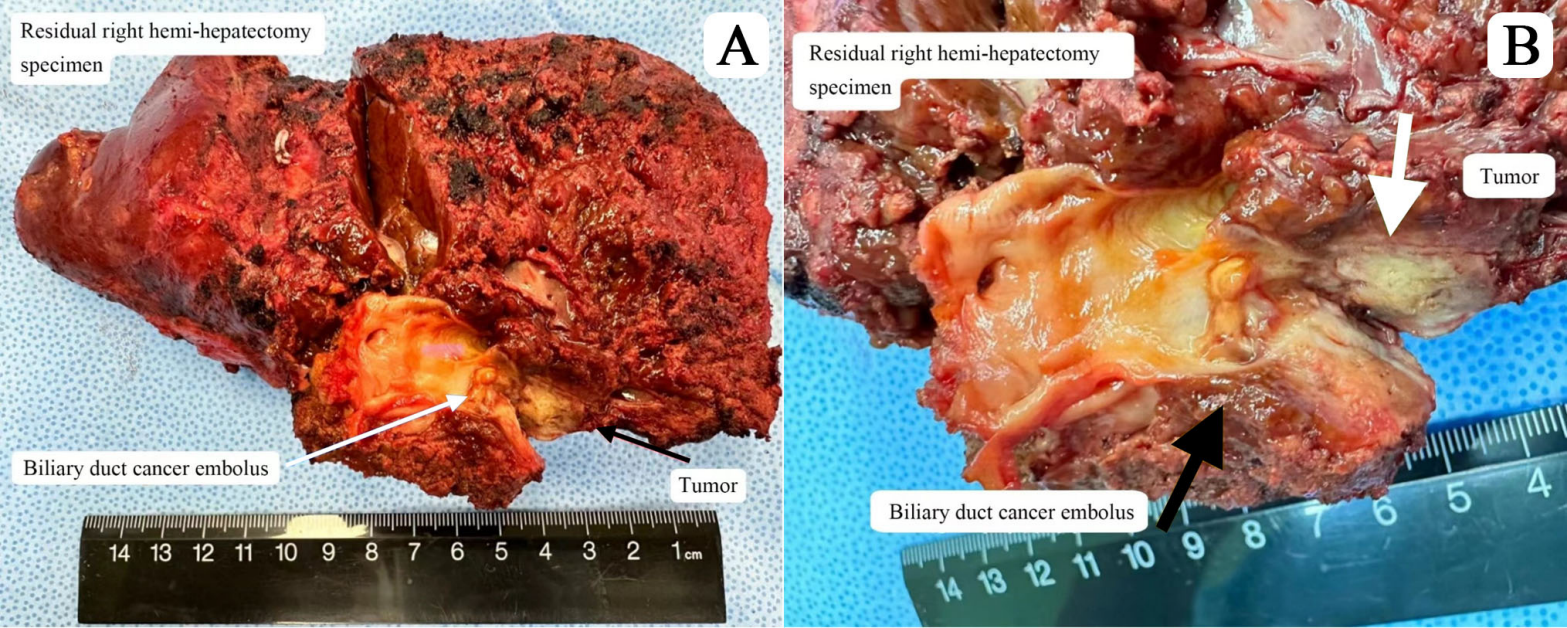
**(A)** Residual right hemi-hepatectomy specimen **(B)** Cut surface of tumor lesion showing biliary duct cancer embolus.

Postoperative pathology confirmed that the tumor was ICC, measuring 1.8 x 1.2 x 0.6 cm (**Figure 3**). The cut surface of the tumor was yellow-white with extensive areas of necrosis (**Figure 3**). After systemic therapy, residual lesions accounted for approximately 70% of the tumor bed. Immunohistochemical analysis showed positive staining for AE1/AE3, CK7, Ki67 (10+%), and PD-L1.

At the one-month postoperative evaluation tumor-specific markers returned to baseline levels. The therapeutic regimen continued with the integrated approach of targeted and immunotherapy. The patient underwent periodic follow-up examinations every three months. At one year postoperatively, a PET-CT examination was performed (**Supplementary Figure 1**). As of now, the patient is alive and has not experienced any recurrence or metastasis 19 months after surgery.

## Discussion

ICC is a rare and aggressive hepatobiliary malignancy, often diagnosed at advanced stages, which contributes to its poor prognosis. Even among patients with resectable ICC, the risk of postoperative recurrence remains significant, with reported recurrence rates exceeding 50% within 2 years (3). While repeat surgery for recurrent resectable ICC may improve survival outcomes, the evidence supporting surgical intervention in this subset of patients remains limited, and standardized therapeutic guidelines are lacking (10).

This case report presents a 62-year-old male with recurrent ICC who achieved prolonged overall survival time (72 months) through a multimodal approach combining targeted therapy, immunotherapy, and sequential surgical resections. The success of this case underscores the potential of integrating molecularly guided systemic therapies into perioperative management, particularly for patients with actionable mutations such as FGFR2 fusion mutation.

Current guidelines do not recommend neoadjuvant therapy for resectable ICC due to insufficient evidence. However, emerging data from studies on advanced ICC suggest that preoperative systemic therapy may downstage tumors and reduce micro-metastatic burden. In this case, we opted for a combination of immunotherapy and targeted therapy rather than conventional chemotherapy. This decision was driven by two considerations. Firstly, the synergistic potential of combining FGFR inhibition with immune checkpoint blockade. Preclinical studies indicate that aberrant FGFR signaling promotes an immunosuppressive tumor microenvironment by upregulating PD-1 expression and enhancing regulatory T-cell (Treg) survival via STAT5 phosphorylation (11, 12). Besides, chemotherapy is associated with a prolonged duration of efficacy and relatively significant side effects; therefore, we did not choose it as the initial treatment, reserving it for use as a second-line option after the failure of targeted therapy and immunotherapy.

Clinical studies demonstrate that combining targeted therapy with immune checkpoint inhibitors significantly improves prognosis in FGFR2-altered ICC patients (13). This aligns with the FIGHT-202 trial which was a pivotal phase II study of pemigatinib in locally advanced/metastatic ICC which reported a 35.5% objective response rate (ORR) in unresectable FGFR2-aberrant cases (12). However, two critical distinctions emerge: (1) FIGHT-202 excluded resectable patients, whereas our case achieved 30% tumor regression through preoperative pemigatinib, enabling curative resection; (2) we combined immunotherapy with FGFR inhibition, a strategy not explored in the trial. These differences may explain our patient’s exceptional 72-month overall survival (OS), tripling FIGHT-202’s median OS of 21.1 months (12).

The observed efficacy may be attributed to immunotherapy’s ability to reverse FGFR-driven immunosuppression by modulating the tumor microenvironment (14). Post-treatment imaging revealed partial regression and resolution of intraductal carcinoma emboli, with no recurrence at 24-month follow-up—surpassing the 19-month median disease-free survival in surgical cohorts (15). This contrasts starkly with French Liver Research Society (AFEF) data showing <21-month OS in unresectable ICC (15), underscoring the potential of multimodal therapy in operable cases. Our findings suggest preoperative FGFR-2 inhibition combined with immune modulation could redefine neoadjuvant paradigms for targetable ICC.

ICC is recognized for its aggressive nature, typically presenting at an advanced stage upon diagnosis, and is associated with a high recurrence rate following surgical intervention. Although radical surgery remains the only curative approach for ICC, recent advancements in targeted therapy research have introduced new perspectives into the diagnostic and therapeutic strategies for ICC (16). This case presents a rare instance of a patient with recurrent ICC who, after undergoing radical surgery, exhibited tumor regression following a comprehensive treatment regimen predominantly featuring targeted therapy, prompted by the detection of an FGFR-2 mutation. The patient subsequently underwent a second radical surgery and continued with targeted therapy postoperatively. To date, follow-up has revealed no signs of tumor recurrence. Our team believes that the success of this case could serve as a clinical foundation for perioperative comprehensive management includes long-term postoperative prevention in future for ICC patients, particularly those with identified targetable mutations.

This study has several limitations. First, the single-patient design precludes definitive conclusions about treatment efficacy. Second, the retrospective nature and lack of post-treatment imaging data (due to patient compliance issues) limit mechanistic correlations which could have provided a more comprehensive understanding of the treatment effects in this specific group of patients.

## Conclusion

Our study underscores the potential use of pemigatinib as a viable preoperative systemic treatment option for ICC with FGFR2 mutation. Our findings demonstrate positive outcomes and tolerance, especially in this patient with recurrent ICC. Nonetheless, future studies are needed to reinforce its applicability for patients with ICC.

## Data Availability

The raw data supporting the conclusions of this article will be made available by the authors, without undue reservation.

## Supplementary material

Supplementary Figure 1 Positron emission tomography-computed tomography (PET/CT) scan. No suspicious primary lesion recurrence in the liver after surgery. Neither lymph nodes nor remote organ metastases.
