# Supplementary figures and images for: Systemic therapy with pemigatinib and sintilimab followed by resection for recurrent FGFR-2-positive intrahepatic cholangiocarcinoma: a case report
Source: Front Oncol. 2025 Apr 4;15:1527372. doi: 10.3389/fonc.2025.1527372 (PMC12006668; doi:10.3389/fonc.2025.1527372)

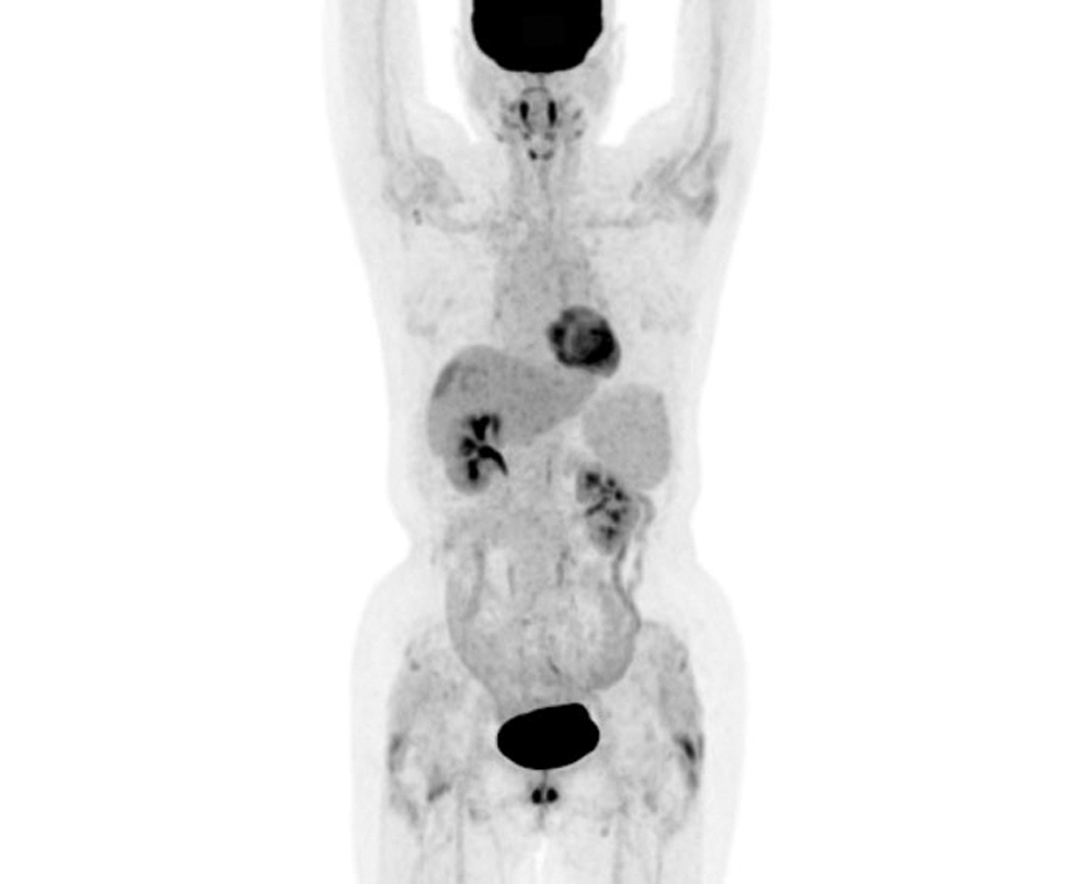

Supplement: Supplementary Figure 1 — Positron emission tomography-computed tomography (PET/CT) scan. No suspicious primary lesion recurrence in the liver after surgery. Neither lymph nodes nor remote organ metastases. [file Image1.jpeg]
