# Supplementary material for: Systemic therapy with pemigatinib and sintilimab followed by resection for recurrent FGFR-2-positive intrahepatic cholangiocarcinoma: a case report
Source: Front Oncol. 2025 Apr 4;15:1527372. doi: 10.3389/fonc.2025.1527372 (PMC12006668; doi:10.3389/fonc.2025.1527372)
Supplement: Supplementary file 2 [file Table1.docx]

**Supplemental Table 1.** Patient characteristics during systemic therapy

| **Characteristics** | Pre-treatment | 1^st^ Post-treatment | 2^nd^ Post-treatment | 3^rd^ Post-treatment | 4^th^ Post-treatment |
| --- | --- | --- | --- | --- | --- |
| WBC, 10^9/L | 4.62 | 5.47 | 4.17 | 4.28 | 4.4 |
| Hb, g/L | 121 | 115 | 122 | 124 | 113 |
| PLT, 10^9/L | 200 | 155 | 178 | 217 | 212 |
| ALB, g/L | 38 | 37 | 43 | 41 | 39 |
| CA199, U/mL | 12.4 | 6.1 | 7.0 | 8.8 | 7.6 |
| CA125, U/mL | 6.0 | 6.5 | 5.5 | 4.5 | 4.3 |
| AFP, ng/ml | 2.64 | 2.28 | 2.65 | 2.84 | 2.27 |
| PVIKA-II | 51.77 | 27.03 | 26.02 | 24.65 | 25.02 |
| CEA, ng/ml | 0.54 | 0.85 | 0.89 | 0.99 | 1.09 |
| TB, μmol/L | 11.8 | 4.7 | 7.9 | 9.1 | 11.1 |
| DB, μmol/L | 2.5 | 1.2 | 0.9 | 1.4 | 1.9 |
| AST, IU/L | 23 | 23 | 20 | 20 | 23 |
| ALT, IU/L | 21 | 12 | 11 | 11 | 13 |
